# Spindle function and Wnt pathway inhibition by PBX1 to suppress tumor progression via downregulating DCDC2 in colorectal cancer

**DOI:** 10.1038/s41389-023-00448-4

**Published:** 2023-02-04

**Authors:** Weigang Dai, Yinan Liu, Tianhao Zhang, Zhixin Huang, Xiang Xu, Zeyu Zhao, Jianqiu Liu, Ertao Zhai, Shirong Cai, Jianhui Chen

**Affiliations:** 1 Division of Gastrointestinal Surgery Center, the First Affiliated Hospital of Sun Yat-sen University, 510080 Guangzhou, Guangdong P. R. China; 2 Gastric Cancer Center, Sun Yat-sen University, 510080 Guangzhou, Guangdong P. R. China; 3 Laboratory of Surgery, the First Affiliated Hospital of Sun Yat-sen University, 510080 Guangzhou, Guangdong P. R. China

**Keywords:** Cancer genetics, Colorectal cancer

## Abstract

PBX1 is a transcription factor that regulates a variety of genes, involved in intracellular lipid metabolism, cell proliferation, and other pathways. The promoting and inhibiting function of PBX1 in various cancer types was extensively discussed, however, there have been no studies on PBX1 proteins in colorectal cancer (CRC). This study aimed to reveal the anti-tumor function of PBX1 in CRC and the underlying molecular mechanism. Bioinformatics analysis revealed that PBX1 is downregulated in CRC, indicating that is a potential antioncogene in CRC. Overexpression of PBX1 suppresses tumor growth and metastasis in vitro and in vivo. Mechanistically, we found that PBX1 acted as a transcription factor that suppressed *DCDC2* expression and inhibited spindle function. Moreover, the PBX1-DCDC2 axis controlled the Wnt pathway in CRC cells. Overexpression of DCDC2 restored CRC proliferation, metastasis abilities and Wnt pathway. In conclusion, this study suggests that PBX1 acts as a transcription factor to suppress DCDC2 expression and inhibit cell proliferation and metastasis by disrupting spindle function and the Wnt pathway in CRC.

## Introduction

Colorectal cancer (CRC) is a serious threat to human health. According to the latest analysis from the World Health Organization, 1,148,515 new colon cancer patients and 732,210 new rectal cancer patients were diagnosed in 2020, ranking fifth and eighth, respectively, in the number of new cancer cases. Meanwhile, 576,858 and 339,022 people died from colon and rectal cancer, respectively, accounting for 9.2% of cancer-related deaths worldwide [1]. CRC remains an important risk factor that threatens human life and health.

Pre-B-cell leukemia transcription factor1 (PBX1) is an important member of the PBX family [2, 3]. In eukaryotic cells, PBX1 binds to the presequenceprotease1 (PREP1) protein or the Meis homobox 1 (MEIS1) protein to form a heterodimer. Due to the nuclear localization signal (NLS) of the PBX1 protein, PBX1-PREP1/MEIS1 dimers regulate downstream gene transcription [4]. On the other hand, PBX1 acts as a co-factor for HOX protein to enhance its transcriptional regulation function [5]. The oncogene function of the PBX1 protein was first reported in acute lymphoblastic leukemia, where t (1:19), a translocation mutation event, resulted in the fusion of the E2A and PBX1 genes and enhanced PBX1 expression [6]. Wang et al. reported that transcription of lipid metabolism-related genes by PBX1 leads to disorders in intracellular lipid metabolism in breast cancer cells, thus affecting the progression of estrogen receptor-negative breast cancer [7]. Furthermore, polypeptides designed according to the sequence of PBX1 have been reported as a possible treatment option for metastatic colon cancer by inhibiting the PBX1-binding protein, HOXB7 [8]. However, there have been no studies on the role of PBX1 in colorectal cancer progression.

Double cortin domain-containing protein 2 (DCDC2) has been reported as a novel regulator of spindle function and the Wnt pathway in renal tubular epithelial cells. DCDC2 participates in the Wnt pathway by binding to the Wnt pathway regulatory proteins disheveled segment polarity protein 2 (DVL2) and DVL3 [9]. Although there have been studies on the influence of DCDC2 methylation on the prognosis of hepatocellular carcinoma (HCC) patients and paclitaxel resistance [10, 11], no evidence has confirmed the biological role of DCDC2 in colorectal cancer or the correlation between DCDC2 and the Wnt pathway in tumors. The Wnt pathway is activated in the vast majority of patients with cancer growth [12, 13]. Approximately 70% of colorectal cancer cases are initiated by mutations in the tumor suppressor gene APC, which lead to the occurrence of benign adenomas in the intestine, and 15% of adenomas progress to malignant colorectal tumors within 10 years [14]. APC gene mutations lead to the activation of Wnt pathway in intestinal epithelial cells, and the activated Wnt pathway is of great significance for colorectal cancer cell proliferation and stem cell maintenance [15]. When the exocrine protein Wnt binds to the amino-terminal of the G-protein-coupled receptor FZD on the surface of the cell membrane, the downstream Dsh protein is activated and phosphorylated. The phosphorylated Dsh protein binds to the gSK-3 β/Axin/APC trimer, leading to a reduction in the degradation of β-catenin protein, which accumulates in the cytoplasm, translocates into a nucleus, and binds to TCF/LEF to promote the transcription of downstream cell cycle disruption related genes [16].

In this study, the biological significance of PBX1 in colorectal cancer was analyzed, and the regulatory role of the PBX1-DCDC2 axis on spindle function and the Wnt pathway was confirmed, providing a theoretical basis for subsequent targeted therapy of CRC.

## Results

### PBX1 was inhibited in colorectal cancer

**Fig. 1:**
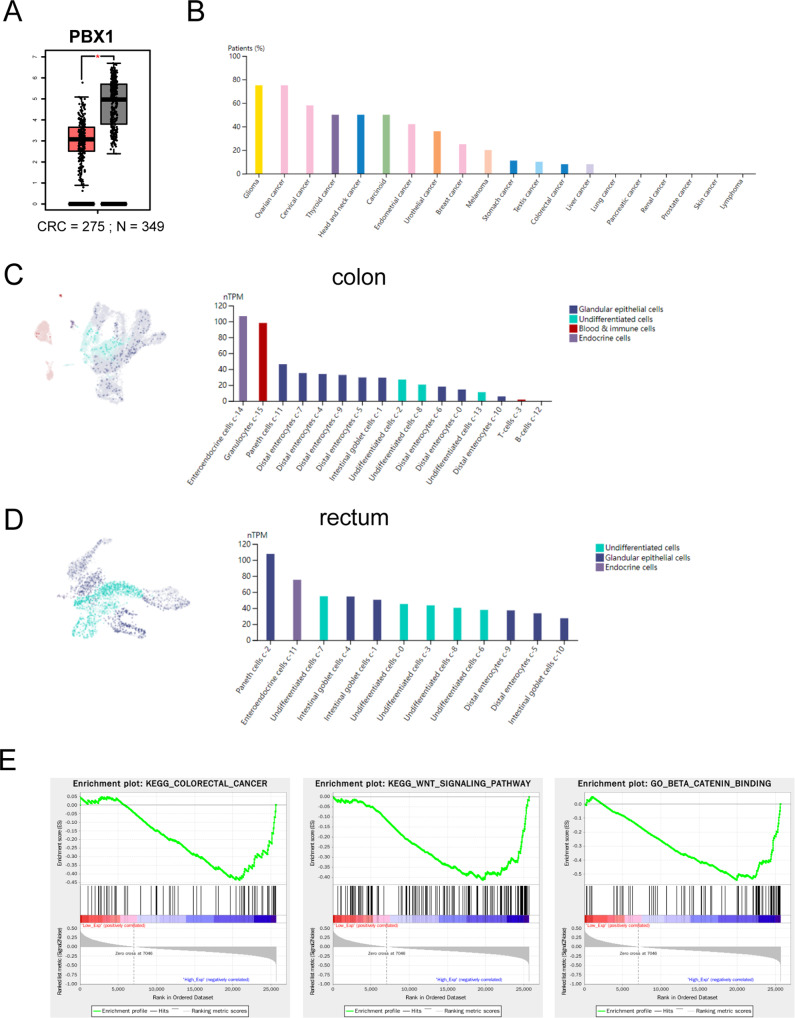
PBX1 expression was suppressed in CRC. **A** The PBX1 RNA levels were lower in CRC samples compared to normal tissues, according to the TCGA and GTEx databases. **B** The expression levels of PBX1 in different cancer types from the THPA database. Both colon and rectal cancer had low levels. **C**, **D** scRNA-seq showed the PBX1 levels in diverse cell types of the colon and rectum. Undifferentiated cells had lower PBX1 expression compared to matured cell types. **E** GSEA analysis indicated that colorectal cancer, Wnt, and beta-catenin pathway were inhibited in PBX1 high group.

PBX1 has been reported to be a transcription factor in many types of cancers, influencing tumor biological behavior and malignant progression. We have previously reported that PBX1 is upregulated in gastric cancer and functions as an oncogene that promotes tumor proliferation and metastasis. However, the PBX1 level was significantly (*P* < 0.05) lower in colorectal tissues than in normal tissues, combining the RNA profile data from TCGA and GTEx databases (Fig. 1A). Furthermore, we explored the expression data of PBX1 in different tissues and organs using the Human Protein Atlas (THPA) database. The order results of PBX1 showed higher expression levels in normal colon tissues than in colorectal cancer tissues (Fig. 1B and Supplementary Fig. S1A). These results suggest that PBX1 expression is suppressed during tumorigenesis. However, K-M curves showed that there was no difference between PBX1 high and low groups on overall survival (OS) in the TCGA database (Supplementary Fig. S1B). This result may because of the interference from tumor stromal cells and infiltrative immune cells on PBX1 levels.

Single-cell RNA sequence (scRNA-seq) data from THPA revealed that PBX1 expression was elevated in mature epithelial cells and reduced in undifferentiated cells in colon and rectum tissues (Fig. 1C, D), indicating a negative association between PBX1 levels and the degree of differentiation. However, this trend was not observed in the small intestine (Supplementary Fig. S1C). Subsequently, we separated the CRC samples in the TCGA database into two groups by PBX1 RNA levels and analyzed the different genes between these groups (Supplementary Fig. S1D). KEGG analysis showed that the different genes were functionally enriched in a series of oncogenic pathways, such as the Wnt, Ras, and PI3K pathways (Supplementary Fig. S1E). GSEA also confirmed that PBX1 negatively correlated with the Wnt pathway (Fig. 1E). Overall, PBX1 is downregulated in colorectal cancer and may function as an antioncogene that controls cell proliferation and stemness.

### PBX1 suppressed proliferation and metastasis of colorectal cancer in vitro and in vivo

**Fig. 2:**
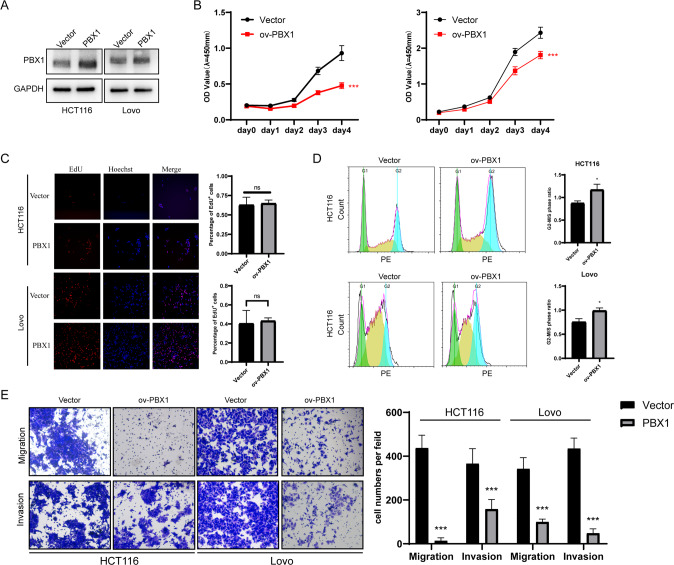
PBX1 suppressed tumor proliferation and metastasis of CRC in vitro. **A** Western blotting confirmed the overexpression efficiency of PBX1 in HCT116 and LoVo cells. **B** PBX1 reduced the proliferative ability of CRC cells as detected by the CCK-8 assay. **C** EdU assay revealed that the percentage of EdU^+^ cells had no effect on PBX1 overexpression. **D** The cells in G2/M phase significantly increased under PBX1 overexpression as measured by flow cytometry. **E** PBX1 suppressed the migration and invasion abilities of CRC as measured by transwell assay. Vector: cells transfected with vector plasmids or lentivirus; ov-PBX1: cells transfected with PBX1 overexpression plasmids or lentivirus.

To investigate the function of PBX1 in colorectal tumors, we chose two cell lines, LoVo and HCT116, which had low PBX1expression levels in CRC cell lines (Supplementary Fig. S2A), to overexpress the PBX1 protein (Fig. 2A and Supplementary Fig. S2B). The CCK-8 assay showed that proliferation ability was significantly decreased upon PBX1 overexpression (Fig. 2B). In contrast, knockdown of PBX1 with specific siRNAs (Supplementary Fig. S2C, S2D) significantly promoted the proliferation of CRC cells (Supplementary Fig. S2E, S2F). Next, EdU analysis was performed to detect the DNA replication rate in PBX1-overexpressed and negative control cells. However, the percentages of EdU-positive cells between the two groups were not significantly different, indicating that DNA replication was not controlled by PBX1 (Fig. 2C). Subsequently, we used cell cycle analysis to explore the stage of cell proliferation that was influenced by PBX1. The percentage of the G2/M phase significantly increased, relative to the other phases, suggesting that there is a G2/M block upon PBX1 overexpression (Fig. 2D). The G2 or mitotic process may be the target of PBX1. Furthermore, we investigated whether PBX1 regulated the metastatic ability of CRC. Transwell analysis confirmed that PBX1 overexpression reduced the migration and invasion abilities of LoVo and HCT116 cells (Fig. 2E), while PBX1 knockdown had the opposite effect (Supplementary Fig. S2G). The effect of PBX1 on chemotherapy in CRC was also investigated. The results showed that there were no significant differences on the susceptibility of cis-platinum or 5-fluorouracil (5-Fu) between PBX1 overexpression and negative control groups (Supplementary Fig. S2H).

**Fig. 3:**
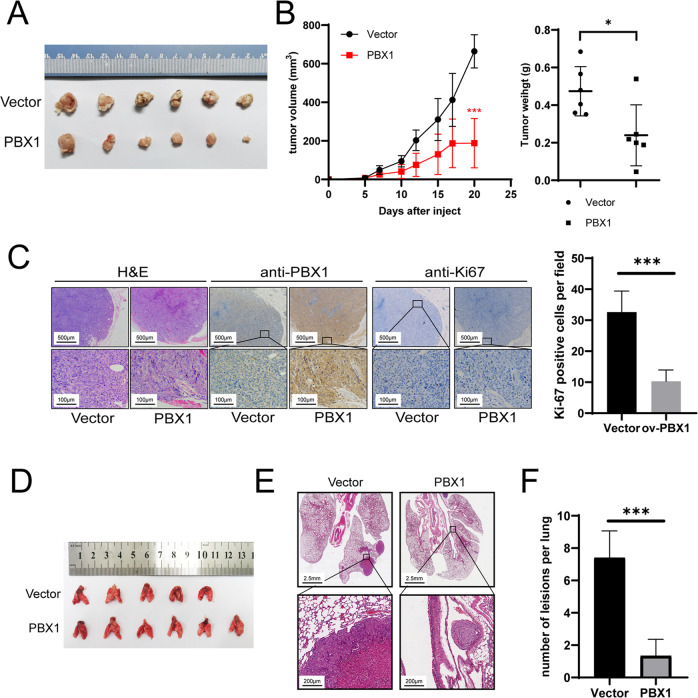
PBX1 inhibited tumor growth and lung metastasis in vivo. **A** The image of xenograft tumors derived from PBX1 overexpression and negative control HCT116 cells. **B** The tumor volume and weight showed the tumor growth of two groups (*n* = 6). **C** Representative H&E and IHC images of PBX1 and Ki-67 in xenograft tumor tissues from (**A**, left panel) and the quantification of Ki-67-positive cells in IHC images (right panel). **D** Lung specimens from lung metastasis model. **E** Representative H&E images showed the lung lesions from lung metastasis model. **F** The number of lung lesions in PBX1 overexpression and negative control groups. Vector: cells transfected with vector plasmids or lentivirus; ov-PBX1: cells transfected with PBX1 overexpression plasmids or lentivirus.

The in vivo effect of PBX1 on cell proliferation and metastasis was also assessed. HCT116 cells treated with stably overexpressed PBX1 and the corresponding control cells were used to construct xenograft models in nude mice. As reflected by tumor volume and weight, PBX1 significantly suppressed tumor growth (Fig. 3A, B). Immunohistochemistry (IHC) images also showed that the proliferation biomarker Ki-67 was less stained in samples with cells overexpressing PBX1 (Fig. 3C). In addition, the overexpression of PBX1 significantly inhibited lung metastasis in vivo, as indicated by hematoxylin and eosin (H&E) staining (Fig. 3D–F). Collectively, PBX1 suppressed CRC tumor proliferation and metastasis both in vitro and in vivo.

### PBX1 suppressed mitosis process and inhibited cell proliferation in intestinal cancer

**Fig. 4:**
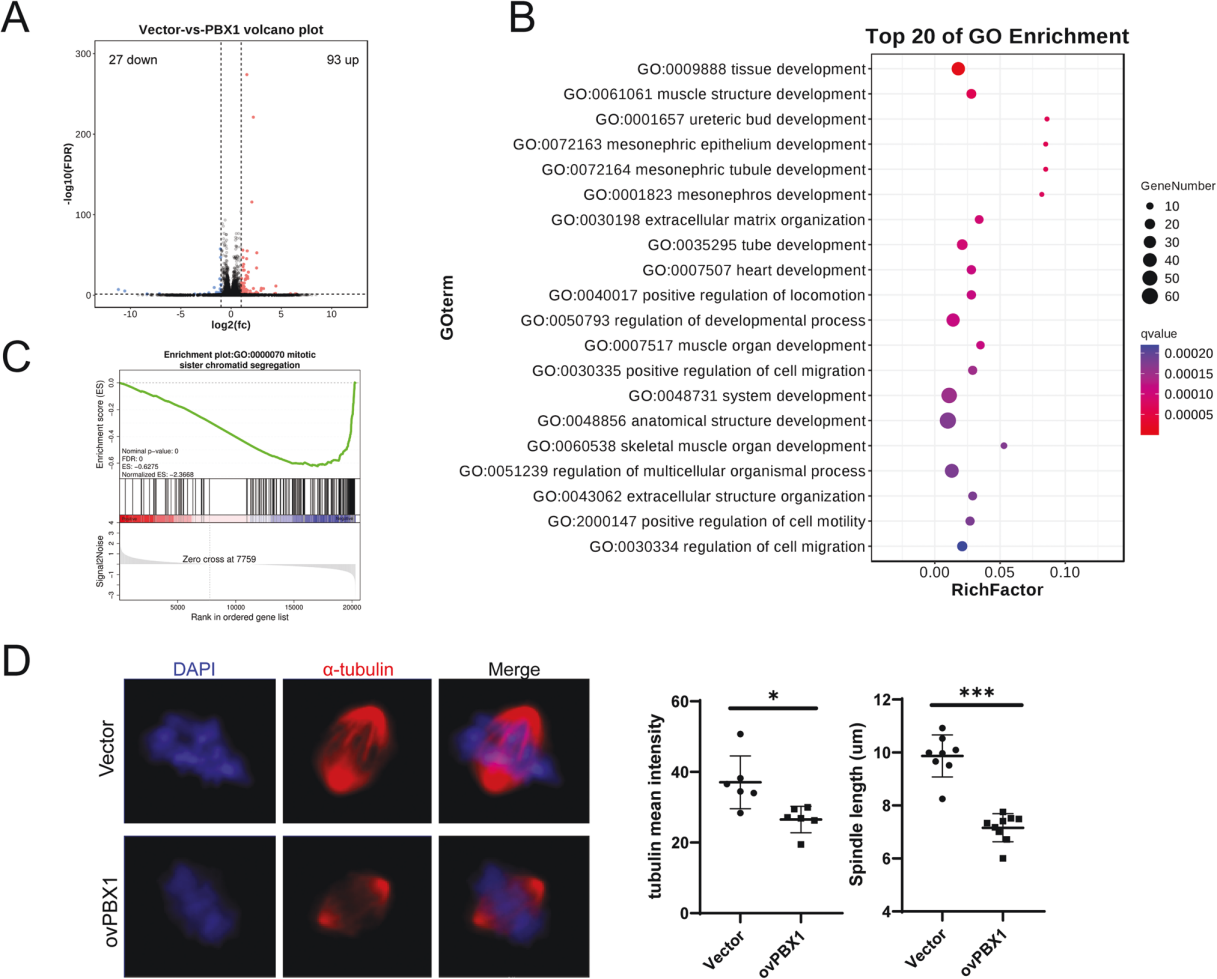
PBX1 suppressed spindle function in CRC cells. **A** The volcano plot showed the differential expression genes between PBX1 overexpression and negative control HCT116 cells according to RNA-seq. **B** The GO enrichment analysis on differentially expressed genes in PBX1 overexpression cells. **C** GSEA analysis confirmed PBX1 inhibited mitotic sister chromatid segregation pathway in CRC cells. **D** Immunofluorescence image showing the spindle in PBX1 overexpression and negative control HCT116 cells. The fluorescence intensity and length of spindle tubulin were measured, respectively.

To investigate the genes downstream of PBX1, the RNA sequencing was employed in cells with overexpressed PBX1 and in the negative control. As shown in Fig. 4A, there were 93 genes upregulated and 27 genes downregulated (|fold change | >2, *P* < 0.05), indicating that the mRNA profile was markedly discrepant upon PBX1 overexpression. KEGG analysis revealed that the differentially expressed genes were enriched in some types of cancer processes and in the pluripotency effectors of the stem cell pathway (Fig. 4B), which was consistent with previously described low levels of PBX1 in undifferentiated enterocytes (Fig. 1C, D). GSEA analysis of different cell cycle phase genes was also performed to determine the precise biological process in the G2/M phase suppressed by PBX1 overexpression. The results showed that the events involved in the mitotic period biological behaviors, sister chromatid segregation, and spindle organization were all inhibited (Fig. 4C and Supplementary Fig. S3A). Microtubulin fluorescence staining images showed that both the length and intensity of microtubulin were decreased, indicating spindle dysfunction during mitosis (Fig. 4D). Furthermore, some dividing cells overexpressing PBX1 showed spindle collapse, as described in a previous study [17], which did not exist in the negative control (Supplementary Fig. S3B). Collectively, PBX1 overexpression reduced mitotic progression and suppressed the proliferation of intestinal cancer cells.

### PBX1 inhibited cancer progression by suppressing DCDC2 transcription

**Fig. 5:**
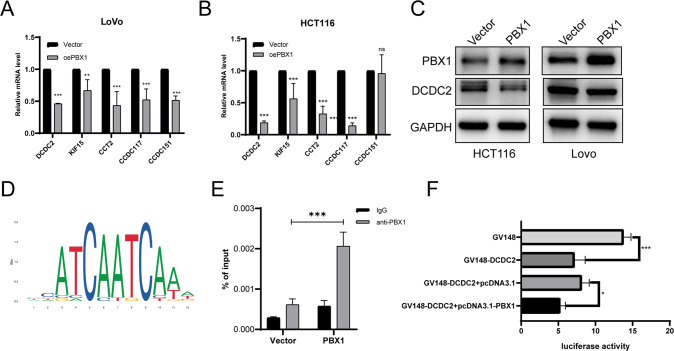
PBX1 transcriptionally suppressed DCDC2 expression in CRC. **A**, **B** RT-qPCR detected the mRNA levels of five potential downstream genes under PBX1 overexpression. **C** Western blotting assay suggested DCDC2 protein levels were decreased under PBX1 overexpression. **D** The predictive sequence of the PBX1-binding site from JASPR database. **E** ChIP-qPCR assay with anti-PBX1 and negative IgG showed that anti-PBX1 enriched the DCDC2 regulator region. **F** The regulator region of DCDC2 was synthesized for the luciferase reporter assay. This region significantly decreased luciferase activity, while PBX1 enhanced this effect.

It was extensively reported that PBX1 worked as a transcription factor regulating downstream gene expression [4, 6, 7]. Therefore, we visited the ChIP-Base v2.0 database [18] to explore the potential genes that bind with the PBX1 protein directly, as reported by Chromatin Immunoprecipitation (ChIP) sequence data from other studies. A total of 103 genes were directly bound with the PBX1 protein in the LoVo cell lines (Supplementary Table S1). Five of these genes, including DCDC2, KIF15, CCT2, CCDC117, and CCDC151, have been reported to be involved in microtubulin polymerization or the spindle organization pathway. Here, we used RT-qPCR analysis to detect the shift in the RNA levels of the five genes upon PBX1 overexpression. The results showed that DCDC2 had the highest stability and the most prominent gene suppression upon PBX1 overexpression (Fig. 5A, B). Western blot analysis also confirmed the downregulation of DCDC2 upon PBX1 overexpression (Fig. 5C). To explore whether PBX1 regulates DCDC2 expression in CRC of clinical patients, the RNA profiles from TCGA database were employed, and negative correlation between these two genes was verified (Supplementary Fig. S4A).

Next, JASPR online tools [19] were used to predict the direct interaction sites between PBX1 and the DCDC2 regulatory region, which are indicated in ChIP-Base v2.0. The results showed that there were several binding sites in the sequence located 1000–10 bp downstream of the transcription start site in *DCDC2* gene (Supplementary Fig. S4B). Considering that DCDC2 levels decreased upon PBX1 overexpression, this region may be the transcription suppressor (TS) of the *DCDC2* gene. ChIP-qPCR was performed to verify the binding of PBX1 to the DCDC2 promoter region. As shown in Fig. 5E, the PBX1 antibody was significantly enriched in the DNA fraction of the DCDC2 regulatory region. The enrichment score was much higher in PBX1 overexpressed cells than in the control cells. Finally, a luciferase reporter assay was conducted to determine whether PBX1 binding to the DCDC2 promoter region suppressed DCDC2 transcription. We found that sub-cloning the sequence of the 1000–10 bp region downstream of the transcription start site of DCDC2 inhibited luciferase activity, which confirmed that this region functioned as a TS. Furthermore, PBX1 overexpression significantly inhibited luciferase activity in the DCDC2 TS region (Fig. 5F).

### Elevated DCDC2 rescued colorectal cancer progression upon PBX1 overexpression

**Fig. 6:**
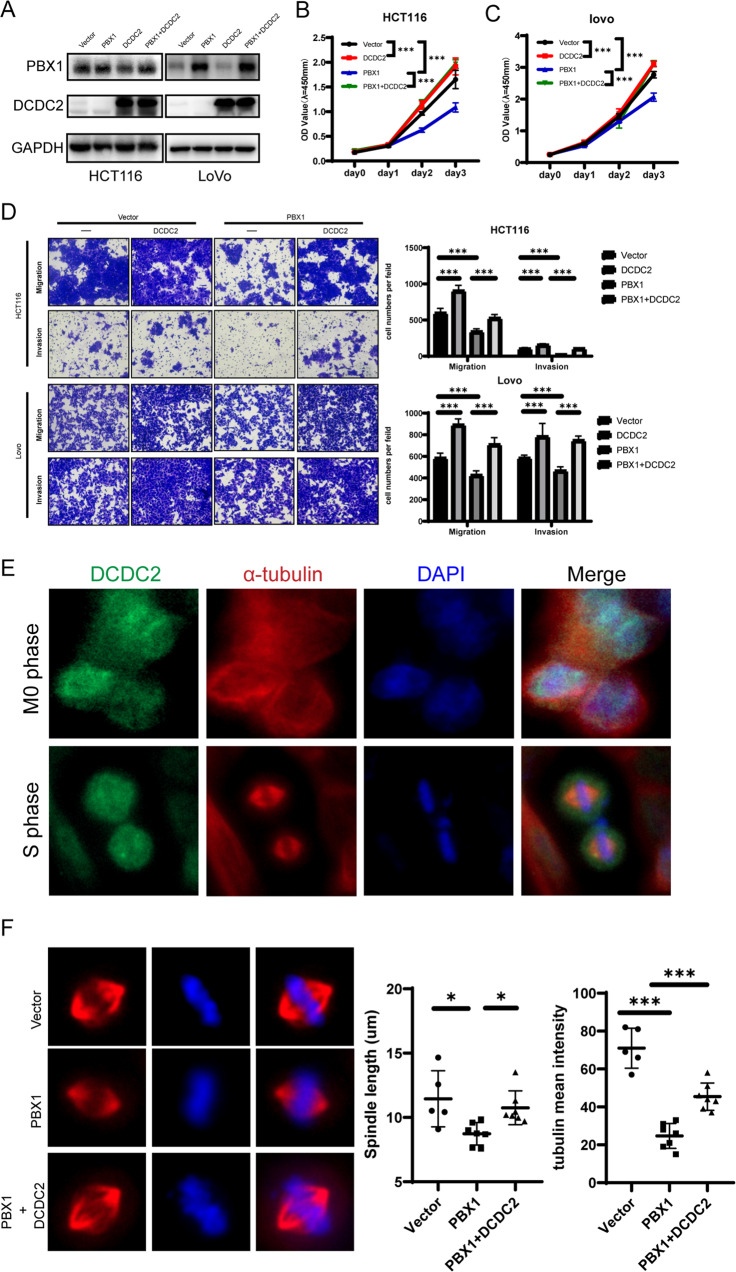
DCDC2 rescued the proliferation and reversed metastasis inhibition under PBX1 overexpression. **A** DCDC2 plasmid was transfected in PBX1 overexpression CRC cells. PBX1 and DCDC2 protein levels were detected by western blotting assay. **B**–**D** The inhibited proliferation, migration, and invasion abilities by PBX1 were rescued upon DCDC2 overexpression as reflected by CCK-8 and transwell assay. **E** Immunofluorescence image of PBX1 and α-tubulin distribution in M0 and S phase of HCT116 cells. **F** The spindle length and tubulin intensity was rescued upon DCDC2 overexpression.

DCDC2 has contrasting effects on tumor progression in diverse cancer types. To verify the effects of the DCDC2 protein in colorectal cancer, we transfected DCDC2 expression plasmids into HCT116 cells (Supplementary Fig. S5A). CCK-8 and transwell assays confirmed that DCDC2 promoted colorectal tumor cell proliferation and metastasis (Supplementary Fig. S5B, S5C). Next, we used DCDC2 expression vectors to rescue DCDC2 protein levels in PBX1 overexpressed cells, and detected the expression efficiency by western blotting (Fig. 6A). Functional cell assays revealed that DCDC2 overexpression dramatically promoted proliferation, migration, and invasion abilities that were inhibited by PBX1 overexpression (Fig. 6B–D).

Moreover, DCDC2 has been reported to bind microtubulin, thereby influencing tubulin polymerization and spindle function. We used immunocytochemistry (ICC) to explore the distribution of DCDC2 and α-tubulin proteins in CRC cells at different cell cycle phases. The images showed that DCDC2 and α-tubulin had limited co-localization in the cytoplasm and nucleus during the interphase. However, in metaphase, DCDC2 and α-tubulin were significantly colocalized in the nucleus, especially in the spindle area (Fig. 6E). ICC analysis also confirmed that DCDC2 overexpression rescued the length and intensity of spindle microtubulin in PBX1 overexpressed cells (Fig. 6F). These results suggest that DCDC2 promotes spindle function and enhances CRC cell proliferation. However, this mechanism does not explain why PBX1 inhibits the metastatic ability of CRC.

### PBX1-DCDC2 axis controlled the Wnt pathway in CRC cells

Previous results in Fig. 1 show that PBX1 is negatively correlated with the Wnt pathway. Meanwhile, it has been reported that DCDC2 binds to DVL2 and DVL3, two important molecules in the Wnt pathway that transduce Wnt signals to downstream effectors. Therefore, we hypothesized that PBX1 inhibits CRC metastasis by suppressing the function of DCDC2 in the Wnt pathway.

**Fig. 7:**
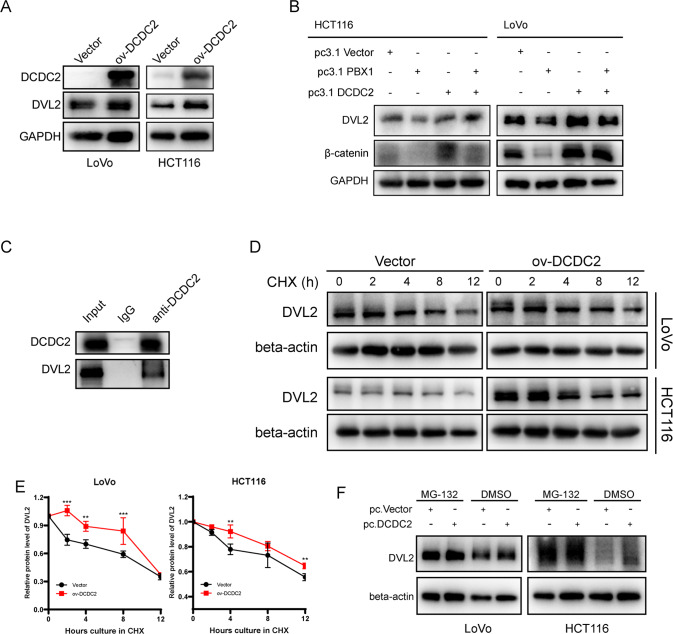
PBX1-DCDC2 axis controlled Wnt pathway in CRC. **A** DVL2 decreased under DCDC2 overexpression as measured by western blotting assay. **B** Western blotting assays detected the DVL2 and β-catenin levels of samples from Fig. 6A. **C** Co-IP experiments indicated that DCDC2 antibody significantly enriched DCDC2 and DVL2 proteins. **D**, **E** CHX analysis explored the degradation speed of DVL2 in DCDC2 overexpression CRC cells. **F** The protein levels of DVL2 in DCDC2 overexpression cells treated with MG-132.

In this study, we transfected CRC cells with DCDC2 plasmids. Western blot analysis showed that DVL2 protein levels were elevated upon DCDC2 overexpression (Fig. 7A). We investigated whether the PBX1-DCDC2 axis affects the Wnt pathway through DVL2. Western blotting was conducted using the samples shown in Fig. 6A to detect DVL2 and β-catenin protein levels in CRC. As shown in Fig. 7B, DVL2 and β-catenin levels were both decreased upon PBX1 overexpression and increased upon DCDC2 overexpression. DCDC2 rescued the DVL2 and β-catenin levels suppressed by PBX1. Thus, the PBX1-DCDC2 axis controlled the Wnt pathway in CRC.

To investigate the underlying mechanism, co-immunoprecipitation (Co-IP) was employed to confirm the direct binding of DCDC2 and DVL2 (Fig. 7C). CHX experiments showed that the overexpression of DCDC2 significantly prolonged the DVL2 half-life in CRC cells (Fig. 7D, E). To explore whether this effect was due to proteasome degradation, we used MG-132, a proteasome inhibitor, to treat DCDC2 overexpressed CRC cells and the corresponding control. Western blot analysis revealed that DVL2 was not increased in DCDC2 overexpressed cells after treatment with MG-132, in contrast to DMSO treatment (Fig. 7F). Taken together, DCDC2 bound to DVL2 protects it from proteasomal degradation to induce the Wnt pathway in CRC.

## Discussion

PBX1 is a canonical transcription factor that controls the progression of various cancers in eukaryotic cells. Our previous study showed that PBX1 promotes proliferation and metastasis in gastric cancer [20]. It has also been reported to act as an oncogene in leukemia [21], breast cancer [7, 22], and ovarian cancer [23, 24]. However, some evidence suggests the opposite function of PBX1 in cancer progression. Thiaville et al. found that some PBX1 target genes, such as FOXO3a and hnRNPUL1, are critical for DNA repair, which may protect cells from random mutations [25]. The co-factor of PBX1, PREP1, functions as a tumor suppressor by improving genome stability [26]. Recently, one study investigated the function of PBX protein family and reported the downregulation of PBX1 and PBX3 and upregulation of PBX4 in CRC. The team also verified the oncopromoter function of PBX4 [27]. The opposite regulation between PBX1 and PBX4 indicated that PBX1 and PBX4 may regulate different downstream genes and have the different function on CRC. Thus, we explored its effect both in vitro and in vivo.

Bioinformatic analysis suggested that PBX1 expression is inhibited by intestinal epithelial cell differentiation. The TCGA database showed that PBX1 levels were dramatically lower in CRC tissues than in normal tissues which was contrary to gastric cancer. Gene function analysis indicated that PBX1 negatively correlated with the Wnt pathway. These results implied a tumor suppressor function of PBX1 in CRC that was inconsistent with most cancer types. However, TCGA analysis showed that CRC patients gained no significant survival benefit from PBX1 high expression. We supposed that the dramatically suppressed of PBX1 in CRC causing that the source of PBX1 mRNA in silico was mainly from tumor stromal cells and infiltrative immune cells so that the PBX1 level in TCGA could not reflect the actual PBX1 expression in tumor cells. However, this hypothesis needs further investigation in the future.

Furthermore, in vitro and in vivo experiments verified the inhibitory effect of PBX1 on tumor proliferation and metastasis in CRC. Mechanistically, we found that PBX1 did not alter the DNA replication rate of CRC, but instead caused G2/M arrest in the cell cycle process. Subsequent RNA-seq analysis indicated that spindle function was impaired by PBX1 overexpression in CRC cells. ICC was conducted in this study to stain the microtubulin of the spindle to confirm its dysfunction in PBX1 overexpressed cells. Thus, we hypothesized that PBX1 suppresses spindle function to inhibit CRC cell proliferation.

A previous study has shown that PBX1 controls the transcription rate of an abundance of target genes that regulate serious biological behaviors [7, 28, 29]. To explore the genes involved in spindle function that are directly regulated by PBX1, we used the CHIP-Base v2.0 database listing all PBX1 binding genes in LoVo cells. Five of these genes have been reported to be associated with tubulin or spindle functions [9, 30–33]. RT-qPCR and western blotting confirmed that DCDC2 was downregulated by PBX1. However, most studies considered PBX1 as a transcription promoter factor [7, 21, 24], which conflicted with our findings. However, there is still evidence suggesting that PBX1 can suppress the expression of target genes [34]. To verify that PBX1 suppressed DCDC2 transcription, ChIP-qPCR and luciferase analyses were performed. The results revealed that PBX1 directly binds to the *DCDC2* regulator region and suppresses *DCDC2* transcription. Given that few studies have reported the regulation of cancer progression by DCDC2, we investigated the effect of DCDC2 overexpression in CRC. As shown in our data, proliferation, migration, and invasion abilities were dramatically enhanced by transfection with DCDC2 expression plasmids. As expected, DCDC2 rescued the malignant phenotype and spindle function that were suppressed by PBX1. Therefore, we hypothesized that PBX1 suppresses DCDC2 transcription to inhibit CRC cell proliferation and metastasis.

However, the mechanism by which PBX1 suppresses DCDC2 and disrupts spindle function explains why PBX1 controls proliferation ability, but not metastasis. There must be other pathways involved in CRC metastasis inhibition by PBX1. To solve this problem, we retrospectively reviewed our data and found that PBX1 expression negatively correlated with the Wnt pathway, which regulates CRC cell metastasis [35]. There is some evidence that DCDC2 interacts with DVL2 to regulate the Wnt pathway [9]. Hence, we hypothesized that the PBX1-DCDC2 axis regulates the Wnt pathway through DVL2. Western blot analysis confirmed the effect of PBX1 and DCDC2 on the Wnt pathway in CRC cells. Co-IP analysis verified a direct interaction between DCDC2 and DVL2 in CRC cells. This interaction increased the stability of DVL2, as demonstrated by the CHX and MG-132 treatment experiments. Because the vital role of DVL2 in the Wnt pathway and the function of the Wnt pathway in CRC metastasis have been widely discussed in previous studies, we did not conduct supernumerary experiments on these subjects in this study.

In summary, our study revealed that PBX1 suppresses the proliferation and metastasis of CRC by decreasing DCDC2 transcription to inhibit spindle function and the Wnt pathway. Nevertheless, due to the limitations of this study, there were important aspects that were not discussed. First, most experiments were performed in PBX1 overexpressed cells without the corresponding knockdown cells. Second, the Wnt pathway could also promote tumor proliferation; however, we did not discuss the interaction between the Wnt pathway and spindle function in CRC proliferation. The molecular mechanism on DCDC2 regulating spindle function in CRC and the crosstalk between DCDC2-spindle and Wnt pathway will be furtherly explored in our further study.

## Materials and methods

### Cell lines, cell culture, and transfection

Human CRC cell lines (HCT116 and LoVo) were purchased from the Shanghai Branch Cell Bank of the Chinese Academy of Sciences (Shanghai, China). Both cells were cultured in DMEM (Gibco, ThermoFisher, Waltham, MA, USA) supplemented with 10% fetal bovine serum (FBS; Biological Industries, Israel) and 1% antibiotics (penicillin/streptomycin, Gibco) at 37 °C with 5% CO_2_. The cell lines were tested for potential mycoplasma contamination by PCR and were confirmed to be mycoplasma-negative. In subsequent experiments, the cells were treated with MG-132 (MedChemExpress, NJ, USA) or cycloheximide (CHX, MedChemExpress) at the indicated concentrations in DMEM with 10% FBS. DMSO was used as a negative control. For chemotherapy susceptibility experiments, cells were treated with cis-platinum (MedChemExpress) for 24 h or 5-Fu (MedChemExpress) for 48 h at indicated concentrations in DMEM with 10% FBS.

siRNA (sequence details in Supplementary Table S2) was transfected using Lipofectamine RNAi Max (Invitrogen, ThermoFisher Scientific, Inc., Waltham, MA, USA) according to the manufacturer’s instructions. Briefly, the appropriate amount of siRNAs was diluted in 100 µL Opti-MEM (Gibco, USA) and mixed with 5 µL Lipofectamine RNAi Max. The mixture was incubated at 25 °C for 20 min and then added to cell culture media. Cells were collected for further experiments after 48 h. The plasmids were constructed using pcDNA3.0. Plasmids were transfected using Lipofectamine 3000 (Invitrogen). Briefly, 150 µL Opti-MEM was used to dilute 2 µg plasmids with 5 µL p3000 and 5 µL lipo3000 separately. The plasmids and lipo3000 solution were mixed and incubated at room temperature for 15 min. The mixture was added to six-well plates, and the cells were collected after 48–72 h.

For lentivirus transfection, the PBX1 overexpression lentivirus and the negative control, purchased from iGene Biotechnology Co., Ltd. (Columbia, MD, USA), were added to cultured cells at MOI = 1:10. Polybrene (5 µL) was added to the medium. Finally, cells were cultured with 2 µg/mL puromycin to select positively transfected cells. The transfection efficiency was detected by qPCR (Primers details in Supplementary Table S3) and western blotting (Antibodies details in Supplementary Table S4).

### CCK-8 assay

Cells were seeded in 96-well plates at 2000 cells/well for the CCK-8 assay. After 0, 24, 48, 72, and 96 h of planting, relative cell numbers were evaluated using 10% CCK-8 (SolarBio, Beijing, China) diluted in standard culture media for 2 h. Quantification was performed on a microtiter plate reader (ALLSHENG, Hangzhou, Zhejiang, China) at a UV wavelength (λ) of 450 nm.

### Cell cycle assay

The cells were cultured in six-well plates at 60–70% fusion degree. Before the experiments, FBS-free medium was added for 12 h to pause the cell cycle. We then used complete media for 8 h and collected cells in pre-cooled 70% ethanol overnight. The cells were then centrifuged at 2000 rpm and washed twice with PBS. Finally, the cells were stained using the Cell Cycle Kit (Yishan, Shanghai, China) for 30 min and detected with CytoFLEX (Beckman Coulter, Brea, CA, USA).

### EdU stain analysis

The population of DNA-replicating cells was assessed using an EdU detection kit (RiboBio, Guangzhou, China). Briefly, the cells were pre-seeded in a 96-well plate for 12 h. Then, EdU staining solution was added to the media and incubated for 2 h. After washing the additional EdU dye, Hoechst stain was used as per the manufacturer’s instructions. Images were obtained using a fluorescence microscope (BX63; Olympus, Tokyo, Japan). The EdU-positive rate was calculated as the ratio of the number of EdU-positive cells to the number of Hoechst-stained cells.

### Transwell analysis

To detect cell migration, 100,000 cells in 400 µL FBS-free media were plated on the top chambers of Transwell clear polyester membrane inserts (Corning Costar, New York, USA). Culture medium containing 10% FBS was applied to the bottom. After 16–72 h, cells were stained with crystal violet and counted under a microscope (Olympus, Tokyo, Japan). For the invasion assay, Transwell clear polyester membrane inserts were pre-coated with 10% Matrigel (Corning Costar) on the top chambers for 2 h. The subsequent steps were the same as those used for the migration analysis.

### Animal models

Specific Pathogen-Free male BALB/c nude mice (5–6 weeks old) were purchased from Biotechnology Co., Ltd. (Beijing, China), and maintained in specific pathogen-free facilities. This study was approved by the institutional ethical board of the First Affiliated Hospital of Sun Yat-sen University. For the tumor xenograft models, 5 × 10^6^ cells were subcutaneously injected into the right axilla of nude mice (*n* = 6 per group). The tumor volume was monitored every other day (volume = length × width^2^ × 1/2). When the largest tumor diameter reached 1 cm, mice were euthanized via cervical dislocation. The tumors were weighed, imaged, fixed in 4% paraformaldehyde, or frozen for further analysis. For the lung metastasis model, 1 × 10^6^ cells were injected intravenously into the tail vein of the nude mice (*n* = 6 per group). After 8 weeks, the mice were euthanized by cervical dislocation, and the lungs were resected, photographed, and fixed in 4% paraformaldehyde for further analysis. One animal of lung metastasis model was excluded because of unexpected death before harvested.

### Immunohistochemistry (IHC)

Formalin-fixed, paraffin-embedded specimens of xenograft models were cut into 3-μm-thick sections and mounted onto adhesion microscope slides. After being deparaffinized, antigen retrieval was performed with an autoclave in 0.01 mol/L EDTA buffer (pH 8.0). Sections were incubated with 3.0% hydrogen peroxide (H2O2) solution for 20 min and then incubated with 5% goat serum diluted in PBST (0.3% Triton X-100 in PBS; Gibco) at 25 °C for 30 min to block non-specific staining. Next sections were incubated with the anti-PBX1 (1:500, rabbit, 18204-1-AP, Proteintech) or anti-Ki-67 (1:500, mouse, 27309-1-AP, proteintech) at 4 °C overnight. After rinsed and sequential 1-h incubations with horseradish peroxidase-conjugated secondary antibody (G1214; Servicebio, Wuhan, Hubei, China), targeted protein was visualized using liquid DAB (Servicebio). Finally, all slides were counterstained with hematoxylin (Solarbio) as instruction.

### hematoxylin–eosin staining (H&E)

The slides were prepared, deparaffinized and hematoxylin stained as described in IHC stain. Then eosin was counterstained for 2 min and washed with ddwater twice.

### RNA sequencing

Total RNA was extracted using Trizol reagent kit (Invitrogen, Carlsbad, CA,USA) according to the manufacturer’s protocol. RNA quality was assessed on an Agilent 2100 Bioanalyzer (Agilent Technologies, Palo Alto, CA, USA). Then, eukaryotic mRNA was enriched by Oligo(dT) beads, while prokaryotic mRNA was enriched by removing rRNA by Ribo-ZeroTM Magnetic Kit (Epicentre, Madison, WI, USA). After fragmented into short fragments, mRNA was reverse transcripted into cDNA with random primers. Second-strand cDNA were synthesized by DNA polymerase I, RNase H, dNTP, and buffer. Then the cDNA fragments were purified with QiaQuick PCR extraction kit (Qiagen, Venlo, The Netherlands), end-repaired, poly(A) added, and ligated to Illumina sequencing adapters. The ligation products were size selected by agarose gel electrophoresis, PCR amplified, and sequenced using Illumina HiSeq2500.

### Spindle function assay

Before the experiments, 10 µmol/L MG-132 was added to the medium for 2 h to block the division of cells in metaphase. The cells were fixed with 4% formaldehyde (Baishi, Tianjin, China), and quenched with glycine (#G8200, Solarbio) at room temperature. The cells were then incubated with diluted 5% goat serum and incubated with α-tubulin antibody (1:500, rabbit, C1050, Beyotime Biotechnology, Shanghai, China) for 2 h at room temperature. The cells were then fixed on the object slide using SlowFade Gold Antifade Mountant (Sigma-Aldrich, Merck Millipore, Darmstadt, Germany). Images were acquired using a BX63 microscope (Olympus).

### Chromatin immunoprecipitation (ChIP)

ChIP was performed using a ChIP assay kit (SimpleChIP® Plus Sonication Chromatin IP Kit (#56383, Cell Signaling Technology, MA, USA). Briefly, 5 × 10^6^ CRC cells were fixed with 1% formaldehyde and quenched with glycine (#G8200; Solarbio) at room temperature. After resuspension in lysis buffer (Cell Signaling Technology, Danvers, MA, USA), the samples were sonicated to break chromatin. Next, the chromatin solution was immunoprecipitated using anti-IgG (DIA-AN, Q6004; Wuhan, Hubei, China) and anti-PBX1 (Abnova, H00005087-M01; Tsukiji, Tokyo, Japan). Immunoprecipitated DNA was purified by column collection and analyzed by qPCR. The enrichment percentage was calculated using the relative quantification method (CT^ChIP − CT^input).

### Luciferase reporter assay

The DCDC2 regulator region was amplified and subcloned into the GV-148 vector (iGene Biotechnology Co., Ltd.) for the luciferase assay. Cell samples were co-transfected with the GV-148 vector and the indicated transfection plasmids for 48 h, and then subjected to the Luciferase Reporter Assay System (Promega, Madison, WI, USA).

### Statistical analysis

Overall survival (OS) is defined as the interval between the first radical resection to the date of death from any cause or to the date of the last follow-up visit. Kaplan–Meier analysis was used to analyze the survival fraction. All statistical values were calculated using Statistical Product and Service Solutions (SPSS) 22.0 (Chicago, IL, USA). The variance similarities between the groups were analyzed by Bartlett test. The data are the means ± SEM of at least three independent experiments. Experimental studies were analyzed by independent sample *t* test (two-tailed) to compare two groups and by one-way ANOVA followed by Tukey’s test to compare multiple groups. Kaplan–Meier analysis and log-rank tests were used to evaluate differences in patient survival. Statistical significance was set at *P* < 0.05.

## Supplementary information

supplemental table S1

Supplemental materials

## Data Availability

The data were uploaded into the GEO database (GSE218378).
