# Supplementary material for: Spindle function and Wnt pathway inhibition by PBX1 to suppress tumor progression via downregulating DCDC2 in colorectal cancer
Source: Oncogenesis. 2023 Feb 4;12(1):3. doi: 10.1038/s41389-023-00448-4 (PMC9899229; doi:10.1038/s41389-023-00448-4)
Supplement: Supplementary file 2 — Supplemental materials [file 41389_2023_448_MOESM2_ESM.docx]

**Supplementary Figures**


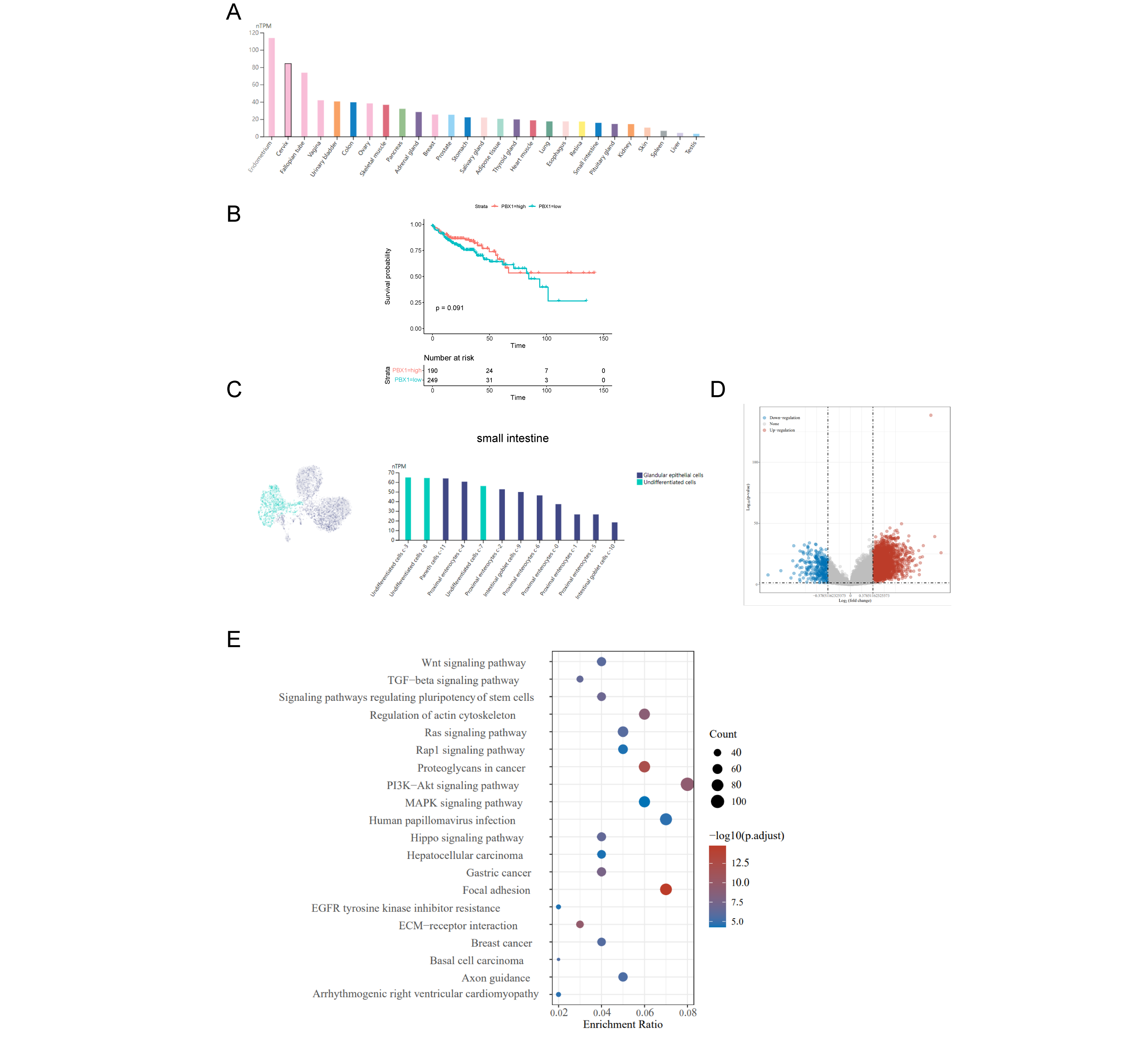


**Supplementary Figure S1. Suppressed PBX1 levels and the potential oncogenesis function in CRC.**

A. The expression levels of PBX1 in different organs or tissues of healthy specimens from THPA database.

B. scRNA-seq showed the PBX1 levels in diverse cell types of small intestine.

C. Kaplan-Meier analysis of overall survival based on PBX1 expression of CRC patients in TCGA database.

D. Volcano plot showed the differently expression genes between PBX1 high and low expression samples from TCGA database.

E. KEGG enrichment analysis identified several cancer related pathways were correlated with PBX1.


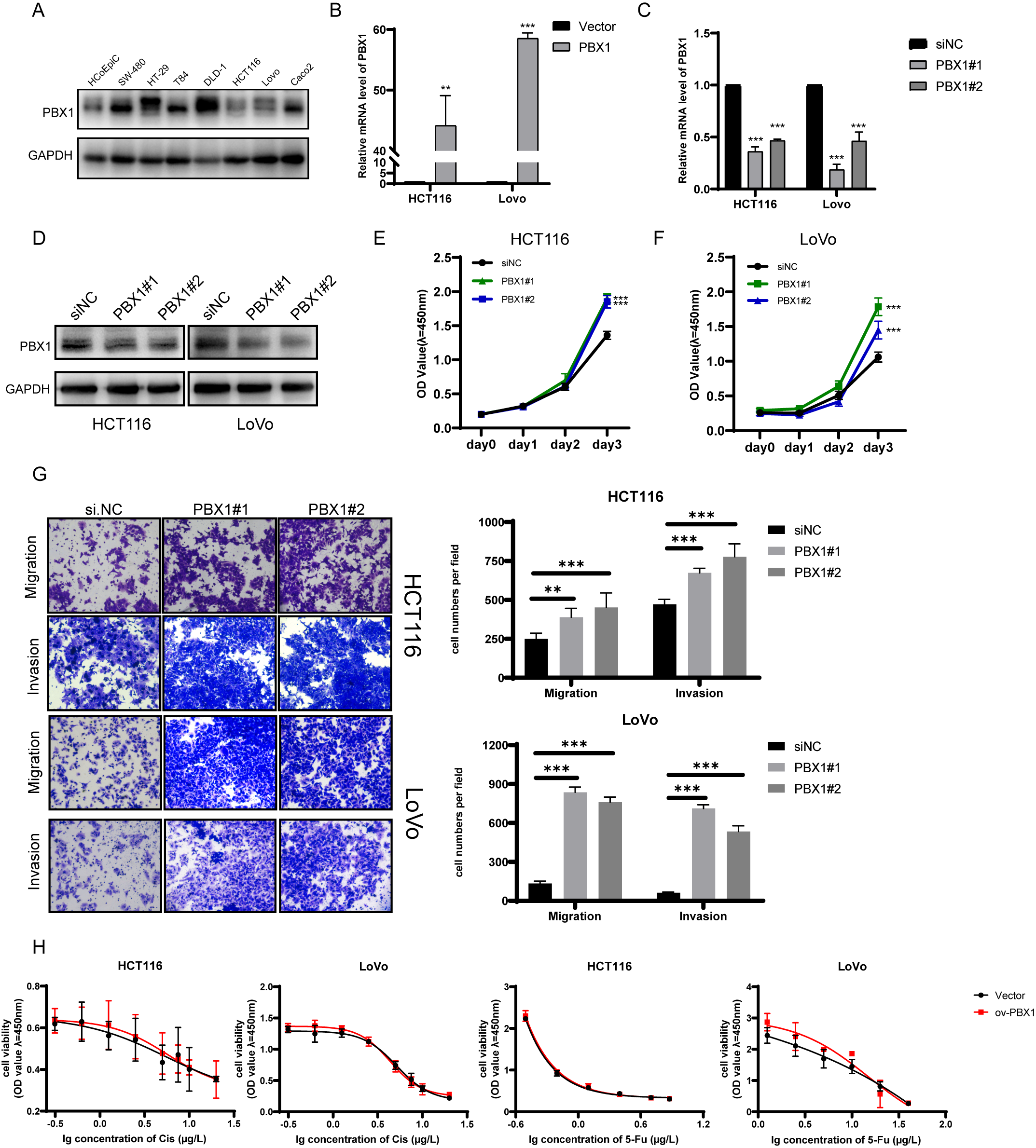


**Supplementary Figure S2. Knockdown PBX1 induced cell proliferation and metastasis ability of CRC in vitro.**

A. Western blotting analysis showed PBX1 protein levels in colorectal epithelial cell and CRC cell lines.

B-C. RT-qPCR measured the efficiency of PBX1 overexpression and knockdown in LoVo and HCT116 cells.

D. Western blotting showed the protein levels of PBX1 in si-PBX1 treated CRC cells.

E-F. Knockdown PBX1 promoted CRC cells proliferation as measured by CCK-8 assay.

G. Knockdown PBX1 promoted CRC cells migration and invasion abilities detected by transwell assay.

H. CCK-8 analysis showed the cell viability of HCT116 and LoVo cells treated with cis-platinum (24h) and 5- fluorouracil (48h) at different concentration.

Cis: cis-platinum; 5-Fu: 5-fluorouracil


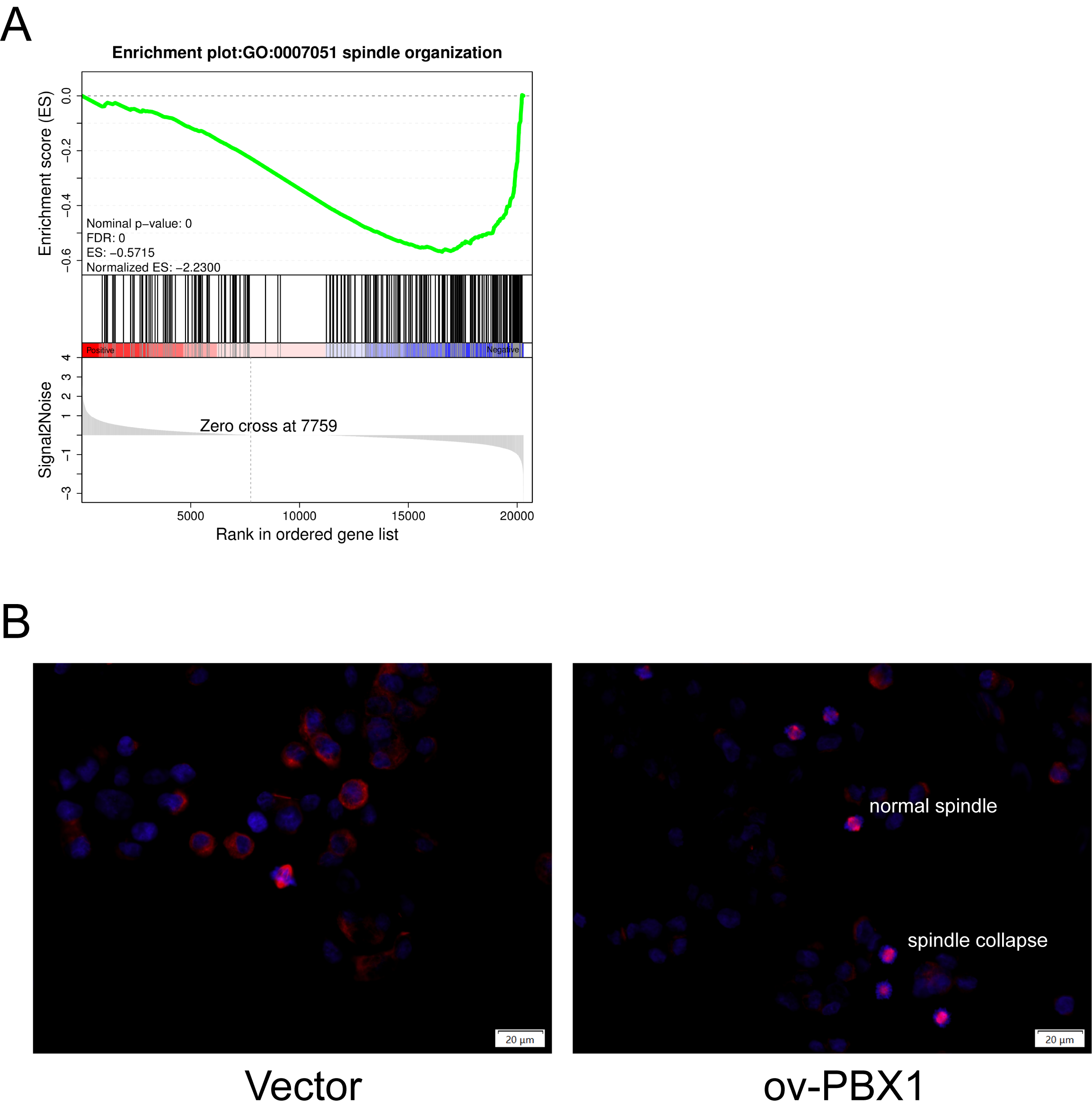


**Supplementary Figure S3. PBX1 inhibited spindle function in mitosis phase of CRC.**

A. GSEA analysis confirmed the spindle organization pathway inhibition in PBX1 overexpression CRC cells.

B. Immunofluorescence image showed the normal spindle in dividing HCT116 cells and spindle collapse in PBX1 overexpressed cells. Blue: DAPI; Red: microtubulin.


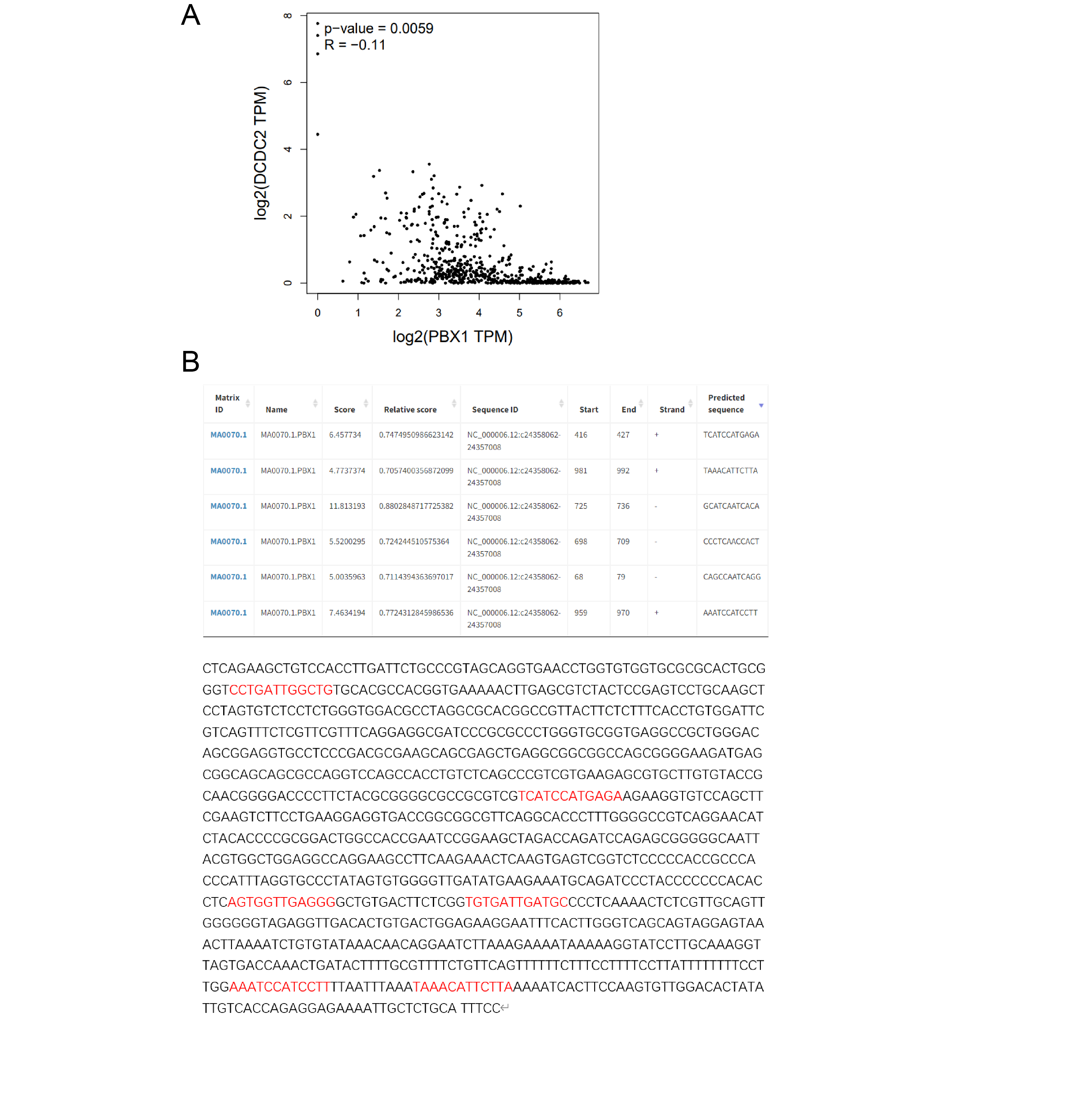


**Supplementary Figure S4. PBX1 working as a transcript factor suppressed DCDC2 transcription.**

A. The RNA levels of PBX1 and DCDC2 in CRC had a negative correlation according to TCGA database.

B. The sequence of predicted region of PBX1 to *DCDC2* gene from ChIP-Base v2.0 database. The red text labeled the possible binding sites calculated by JASPR Scan tools.


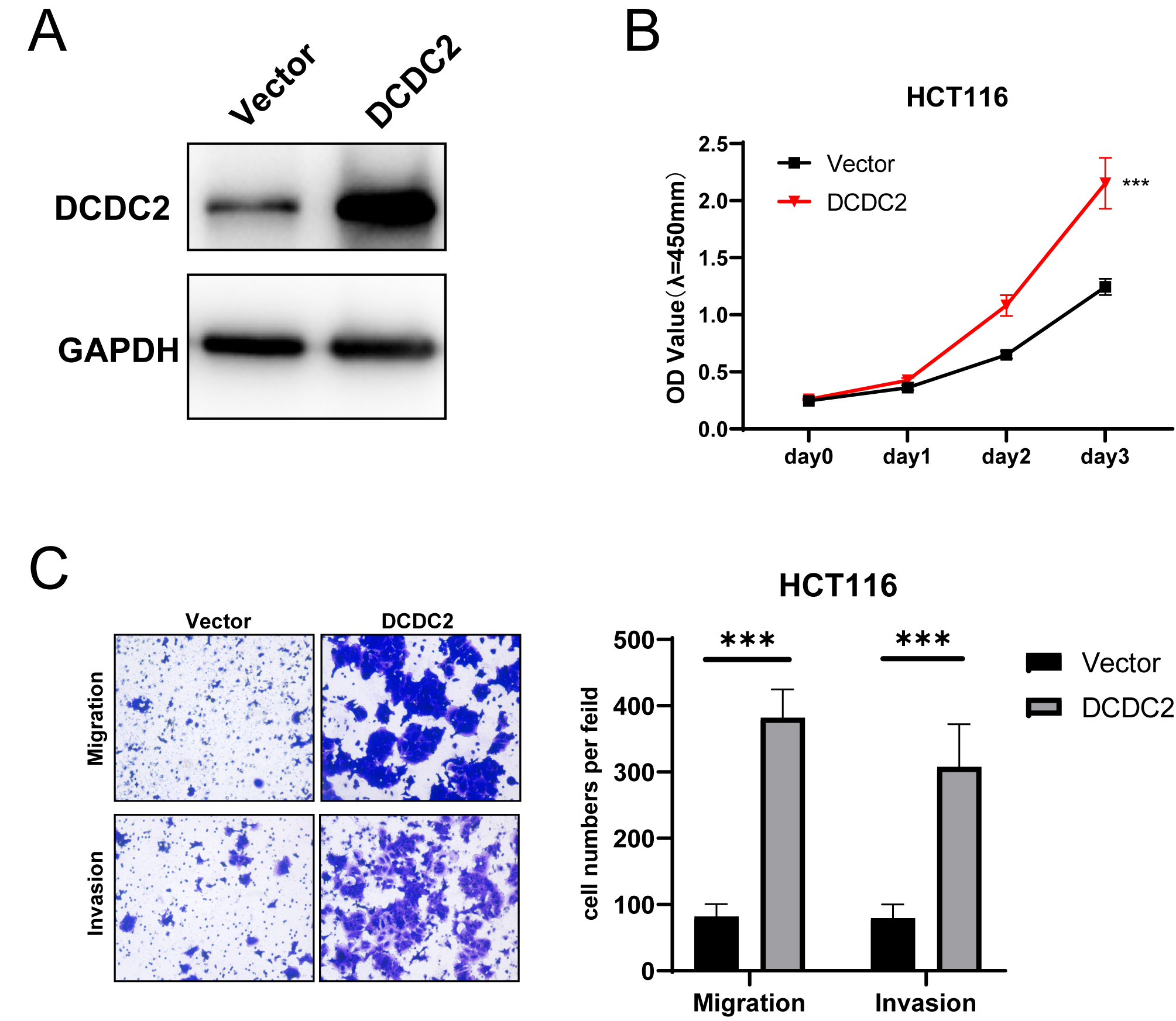


**Supplementary Figure S5. DCDC2 promoted CRC cells proliferation, migration and invasion abilities.**

A. Western blotting verified the expression efficiency of DCDC2 plasmids in HCT116 cells.

B. CCK-8 assay indicated that DCDC2 promoted HCT116 cells proliferate.

C. DCDC2 overexpression accelerated HCT116 cells metastasize.

**Supplementary Tables**

**Supplementary Table S1. The mapped genes of ChIP-seq data of PBX1 in CHIP-Base database.**

(Excel file)

**Supplementary Table S2. Sequences of siRNAs used in this study.**

| **siRNA** | | **Sequences (5’-3’)** |
| --- | --- | --- |
| PBX1#1 | sense | GAGCCAAACUCUCACAGAU dTdT |
|  | antisense | AUCUGUGAGAGUUUGGCUC dTdT |
| PBX1#2 | sense | GUGGAGCAUUCAGAUUACA dTdT |
|  | antisense | UGUAAUCUGAAUGCUCCAC dTdT |

**Supplementary Table S3. Sequences of primers used in this study.**

| **Primer** | | **Sequences (5’-3’)** |
| --- | --- | --- |
| **For RT-qPCR** | | |
| GAPDH | Forward | ACAACTTTGGTATCGTGGAAGG |
|  | Reverse | GCCATCACGCCACAGTTTC |
| PBX1 | Forward | GACAACTCAGTGGAGCATTCA |
|  | Reverse | CTCTCGCAGGAGATTCATCAC |
| DCDC2 | Forward | CCAGCTTCTCGCCTCCTTATC |
|  | Reverse | GGCCTTCTCATCGTTGACTTG |
| KIF15 | Forward | AGGAATCTGTATTCGCAACTGTG |
|  | Reverse | ACTTCGTGGGATTACTCCTCTC |
| CCT2 | Forward | GCACTACCTCTGTTACCGTTTT |
|  | Reverse | CTTCTCTCCAACCCGCTATGA |
| CCDC117 | Forward | CGCGGACGTGTTTCTGTTC |
|  | Reverse | CCAGTCATTAGGACCAGCACA |
| CCDC59 | Forward | CAGAAGACATGGCGGCCTAA |
|  | Reverse | TTCCAGTGACGTTTGAGCCTT |
| CCDC151 | Forward | CTCCGCAAGGAGACTAAGGC |
|  | Reverse | CTCAGCCGGTGGTCTAGGT |
| **For ChIP-qPCR** | | |
| pDCDC2 | Forward | TCTCGTTCGTTTCAGGAGGC |
|  | Reverse | AGACTTCGAAGCTGGACACC |

**Supplementary Table S4. Antibodies used in this study for western blotting, ChIP, and IHC.**

| **Antibody** | **Application** | **Dilution** | **Supplier** | **Catalog number** |
| --- | --- | --- | --- | --- |
| GAPDH | WB | 1/10000 | Bioss | 10900R |
| beta-actin | WB | 1/10000 | DIA-AN | 2060 |
| PBX1 | WB/IHC | WB: 1/1000  IHC:  1/500 | Proteintech | Cat No. 18204-1-AP |
| PBX1 | ChIP | ChIP: 1/50 | Abnova | H00005087-M01 |
| DCDC2 | WB | WB: 1/1000 | Invitrogen | PA5-56787 |
| Beta-catenin | WB | 1/1000 | Proteintech | 17565-1-AP |
| DVL2 | WB | 1/1000 | CST | 3224 |
| α-tubulin | IF | 1/100 | Beyotime Biotechnology | C1050 |
| Mouse IgG | ChIP | 1/1000 | DIA-AN | Q6004 |
| Ki-67 | IHC | 1/500 | Proteintech | Cat No. 27309-1-AP |
| peroxidase-conjugated secondary antibody | IHC | - | Servicebio | G1214 |
